# ASSESSMENT OF SERUM HOMOCYSTEINE, ENDOTHELIN-1, AND NITRIC OXIDE LEVELS IN BEHÇET’S DISEASE

**DOI:** 10.4103/0019-5154.70667

**Published:** 2010

**Authors:** Abeer A Hodeib, Tarek A Elsharawy, Hisham A Fawzi

**Affiliations:** *From the Assistant Professor of Dermatology and Venereology, Tanta University, Egypt*; 1 *From the Lecturer of Internal Medicine, Tanta University, Egypt*; 2 *From the Assistant Professor of Clinical Pathology Department, Alazhar University (males), Egypt*

**Keywords:** *Behçet’s disease*, *homocysteine*, *endothelin-1*, *nitric oxide*

## Abstract

**Background:**

Some prominent features of Behçet’s disease (BD) are arterial and venous thromboses as a result of endothelial dysfunction. Hyperhomocysteinemia is responsible for vascular endothelial injury due to an increased frequency of thrombogenesis. Endothelin-1 (ET-1) is a vasoconstrictor whereas nitric oxide (NO) is an endothelial vasorelaxing peptide that is responsible for the inhibition of platelet adhesion.

**Aim:**

To evaluate serum levels of homocysteine (Hcy) and determine whether hyperhomocysteinemia is considered as a contributing risk factor for venous and arterial thromboses of BD, and to correlate serum levels of ET-1 and NO with disease activity.

**Materials and Methods:**

We measured serum levels of Hcy, ET-1, and nitrite (NO_2_^−^) in 25 patients who fulfilled the criteria of the International Study Group for BD, and compared them to those of 15 healthy control subjects. Levels of Hcy and ET-1 were measured by using enzyme-linked immunosorbent assay (ELISA), whereas serum nitrite (NO_2_^−^) levels were measured by using Griess reaction as an indicator for NO production. All the patients were screened for a history of venous thrombosis and subdivided into thrombotic and nonthrombotic subgroups according to their thrombotic history. Patients with BD were divided into two subgroups, active and inactive, according to their clinical and laboratory findings.

**Results:**

There were significant increases in serum levels of Hcy, ET-1, and nitrite in BD patients compared to those in controls. There was a significant increase in serum Hcy levels in thrombotic compared to nonthrombotic subgroups. Positive correlations were detected between the serum ET-1 and nitrite levels with disease activity in BD patients.

**Conclusion:**

Hyperhomocysteinemia may play some role in the development of venous and arterial thromboses in BD. Increased NO production might ave critical biological activities that are relevant to pathological events in the active period of the disease.

## Introduction

Behçet’s disease (BD), a chronic, progressive, immunoinflammatory disease of unknown etiology, was first described by a Turkish dermatologist, Professor Hulusi Behçet in 1937, as a triad of recurrent oral and genital ulcerations and uveitis. It is now known that BD affects almost every tissue and organ in the body without exception, and its multisystemic involvement may have a poorer prognosis.[1] The etiopathogenesis of BD is probably multifactorial and can involve complex interactions of genetic and environmental factors. There is immunological dysfunction that may be induced by microbial pathogens in genetically susceptible individuals.[2]

Its predominant histopathological features are vasculitis with vessel walls and perivascular infiltration, mainly by lymphocytes, but also by monocytes, plasma cells, and neutrophils; endothelial edema; degeneration at the elastic lamina interna; fibrinoid necrosis; and deposition of immune complex at the vascular wall.[3] Less than half (25–37%) of the patients develop vascular complications during the course of BD. Venous lesions of BD are manifested as superficial thrombophlebitis, and deep vein thromboses of the lower extremities, while the arterial lesions manifested as true and/or false aneurysms and thrombotic occlusion.[4]

Homocysteine (Hcy) is a nonessential, sulfur-containing amino acid formed during the metabolism of methionine to cysteine. Hyperhomocysteinaemia has been regarded as a new modifiable risk factor for atherosclerosis and vascular disease.[5] It enhances lipid peroxidation, vascular smooth-muscle cell proliferation, vascular endothelial injury, increases platelet aggregation, and acts on the coagulation cascade and on fibrinolysis, thereby converting the normal endothelium to a more prothrombotic phenotype.[6]

Endothelin-1 (ET-1) and nitric oxide (NO) are two cellular mediators released from the endothelium. Endothelin-1, an inflammatory mediator, can stimulate macrophages and monocytes to release proinflammatory cytokines.[7] Endothelial dysfunction (ED) is a constant feature of BD.[8] Overexpression of proinflammatory cytokines, adhesion molecules, free oxygen radicals, and high homocysteine levels have been suggested to be responsible for the activation of endothelial cells, irrespective of overt vascular manifestations.[9]

Nitric oxide (NO) is a reactive nitrogen intermediate. It is enzymatically synthesized from L-arginine by a family of isoenzymes of nitric oxide synthase (NOS). It is an important mediator of immunity and inflammation that induces endothelial vasorelaxation and inhibition of platelet adhesion.[10] Based on these data, the present study aimed to evaluate the correlation of Hcy levels with the levels of endothelium-derived ET-1 and NO and disease activity.

## Materials and Methods

Twenty-five BD patients were enrolled in this study (males/females = 15/10). Their age ranged from 20 to 50 years with a mean of 35 ± 6.89 years, and the disease duration ranged from a year to 17 years with a mean of 7.5 ± 1.4 years. The patients were diagnosed on the basis of the criteria defined by the International Study Group for the diagnosis of BD[11] and followed up in the Dermatology and Internal Medicine Departments in Al-Hammadi Hospital, Riyadh, Kingdom of Saudi Arabia. In addition, 15 healthy persons of matched age and sex served as controls.

Clinical and laboratory findings were used to classify the patients as having active or inactive (16/9) disease status. The clinical findings for diagnosis require the presence of recurrent oral ulceration in the form of recurrent minor aphthous, major aphthous or herpetiform ulceration at least three times in a twelve-month period, in addition to any two of the following additional features:

1. recurrent genital ulceration,
2. ocular involvement,
3. skin manifestation that includes erythema nodosum, papulopustular lesions, acneiform nodules, and/or pseudofolliculitis,
4. positive pathergy test,
5. subcutaneous thrombophlebitis, deep vein thrombosis, arterial occlusion or aneurysm,
6. gastrointestinal ulceration,
7. epididymitis,
8. CNS involvement,
9. arthritis, or
10. a family history of the condition.

History, full medical and dermatological examination, and routine laboratory investigations including complete blood count using an automated cell counter, liver and renal function tests, fasting blood sugar, protein, lipid fractions including total cholesterol, triglycerides, high-density lipoprotein (HDL) and low-density lipoprotein (LDL) cholesterol, urinanalysis, and pulmonary functions were done for all the patients and controls. Laboratory investigations were conducted to classify the patients as having active and inactive disease status, including CRP using a sandwich enzyme-linked immunosorbent assay (ELISA) technique, erythrocyte sedimentation rate (ESR) according to the Westergren method, and neutrophil count. All thrombotic events were assessed clinically and confirmed by Doppler sonography, venography, computed tomography, or magnetic resonance imaging.

None of the patients and controls showed any evidence of any other concomitant dermatological or medical disorders such as other autoimmune, atopic disorders, anemia, nutritional deficiency, history of hypertension, and malignancy. Subjects with a history of current smoking, oral contraceptives, vitamin supplementation, or alcohol intake were excluded from the study. None of them had received any topical or systemic medications for at least two weeks before blood collection. All subjects had been informed about the aim and the procedures of the study and had given their consent.

For all patients and controls, the following were done:

Venous peripheral blood samples were drawn in the morning hours between 8:00 and 10:00 AM after overnight fasting. Blood samples were drawn using a 25-gauge needle, avoiding hemolysis, into plain tubes.

### Serum Hcy analysis

As the serum concentration of Hcy increases artificially due to time- and temperature-dependent release from RBCs if blood is stored uncooled and centrifuged immediately after sampling, the blood samples were centrifuged immediately in a cooling centrifuge. Total serum homocysteine (tHcy) levels were determined by using an ELISA Kit (Homocysteine Enzyme Immunoassay Kit, Bio-Rad Lab, Oslo, Norway).

### Serum endothelin-1 (ET-1) analysis

Serum endothelin-1 (ET-1) levels were assayed with a commercially available ELISA Kit (endothelin-1 Enzyme Immunoassay Kit, Cayman Chem, Ann Arbor, MI, USA). This sandwich enzyme-linked immunoassay permits ET-1 measurements with a detection limit of 1.5 µmol/mL.

### Serum nitric oxide (NO) analysis

Serum nitrite levels are a measure of nitric oxide production. Total serum nitrite (NO_2_^−^) levels were measured using the sensitive, colorimetric enzymatic Greiss reagent according to Granger’s method.[12]

There is specificity of serum nitrite as an indicator of basal nitric oxide formation in human forearm circulation. Inhibition of eNOS resulted in a reduction of the basal serum nitrite concentration, which was paralleled by a marked vasoconstriction, suggesting that at least a substantial proportion of the basal nitrite level is attributable to endothelial NO formation.

The serum concentration of nitrite reflects the activity of the endothelial L-arginine–NO pathway.

### Statistical analysis

Data are presented as mean ± SD values. The significance of the mean differences between groups was assessed by Student’s t-test. Relationships among parameters were determined by correlation analyses. *P* < 0.05 were considered to be statistically significant.

## Results

The BD patient group consisted of 15 males and 10 females aged 20–50 years (median: 34, mean: 35 ± 6.89 years) with disease duration between one and 17 years (mean: 7.5 ± 1.4 years). The control group included 15 healthy persons with matched age and sex (ten males and five females) aged 23-48 years (median: 36, mean: 37 ± 8.03 years).

The 25 patients were classified into active or inactive subgroups (16 active patients and 9 inactive patients). Clinical presentations of all patients are reported in Tables 1 and 2. Nine out of 25 patients (36%) were without active involvement.

**Table 1:** Clinical features of patients with Behçet’s disease

	Number of patients (25)	Percentage (100%)
Major criteria		
Oral ulcers	16	64
Genital ulcers	12	48
Anterior uveitis	6	24
Minor criteria		
Pathergy test	2	8
Folliculitis	4	16
Erythema nodosum	1	4
Vascular manifestations	12	48
Inflammatory ocular involvement	10	40
Articular (nonspecific arthritis and synovitis)	4	16
Neurological	2	8
Gastrointestinal	1	4

**Table 2:** Vascular manifestations in Behçet’s disease patients

Vascular manifestations	Number of patients (12)	Percentage (100%)
Venous	8	66.7
Arterial	3	25
Both venous and arterial	1	8.3
Deep vein thrombosis	8	66.7
Superficial thrombophlebitis	4	33.3
Varicose veins	3	25
Pulmonary embolism	1	8.3
Superior venacaval thrombosis	1	8.3

The mean serum Hcy level for all BD patients was 12.8 ± 5.4 µmol/L which was significantly higher than that in the controls (8.5 ± 2.82 µmol/L; *P* < 0.001). There was a statistically significant increase in serum Hcy concentrations in the thrombotic subgroup (40%; mean: 16.3 ± 6.19 µmol/L) compared to those in the nonthrombotic subgroup (6%; mean: 10.5 ± 3.37 µmol/L; *P* < 0.001). There was no significant difference between the serum Hcy concentrations in BD patients who were smokers and nonsmokers (*P* > 0.05).

The mean serum ET-1 level for all BD patients was 25.2 ± 11.35 µmol/mL which was significantly higher than that in the controls (8.75 ± 1.34 µmol/mL, *P* < 0.001). The mean serum ET-1 concentrations in the active and inactive patient groups were 32.85 ± 5.57 µmol/mL and 11.65 ± 1.81 µmol/mL respectively. The difference between the levels in the two groups was statistically significant (*P* < 0.001). The mean serum ET-1 concentration was significantly (*P* < 0.001) increased in the active group patients compared to that of the control group. There was no significant difference between serum ET-1 concentrations in the inactive BD patients and the control subjects.

Serum nitrite levels were significantly increased in the BD patient group compared to the controls (mean: 30.45 ± 10.24 µmol/L, 24.35 ± 5.24 µmol/L, respectively; *P* < 0.001). Serum nitrite levels (mean: 35.25 ± 10.45 µmol/L) were found to be significantly increased (*P* < 0.001) in the active patients’ group compared to the inactive (mean: 46.74 ± 10.62 µmol/L) and the control (mean: 24.35 ± 5.24 µmol/L; *P* < 0.001) groups. The difference between the serum nitrite levels in the inactive patients and the controls was not significant (*P* > 0.05).

There were statistically significant increases in ESR, CRP, and neutrophil counts in active BD patients compared to the inactive (stable) subgroup of BD patients (for each, *P* < 0.01) and the control subjects (for each, *P* < 0.001). The results are explained in Table 3.

**Table 3:** Levels of Hcy, ET-1, and NO in BD patients compared to controls

Parameter	Behçet’s disease	Control	*P* value
Number (M/F)	25 (15/10)		15 (10/5)	
Age (years)	35 ± 6.89		37 ± 8.03	
Hcy (µmol/L)	12.8 ± 5.4			< 0.001
	Thrombotic (40%)	Nonthrombotic (60%)	8.5 ± 2.82	< 0.001
	16.3 ± 6.19	10.5 ± 3.37		
ET-1 (µmol/mL)	25.2 ± 11.35			< 0.001
	Active	Inactive	8.75 ± 1.34	< 0.001*
	32.85 ± 5.57	11.65 ± 1.81		< 0.001**
				> 0.05***
Nitrite (µmol/L)	30.45 ± 10.24			< 0.001
	Active	Inactive	24.35 ± 5.24	< 0.001*
	35.25 ± 10.45	46.74 ± 10.62		< 0.001**
				> 0.05***
ESR (mm/h)	Active	Inactive	8.68 ± 1.41	< 0.001*
	30.84 ± 8.25	17.54 ± 5.76		< 0.001**
CRP (mg/dL)	Active	Inactive	1.84 ± 1.55	< 0.001*
	12.20 ± 10.55	3.40 ± 5.75		< 0.001**
Neutrophil count (10³/mL)	Active	Inactive	2.55 ± 0.35	< 0.01*
	5.70 ± 1.43	4.55 ± 0.66		< 0.001**

Correlation between the patient and the control groups (*P*)

Correlation between the active and the inactive stage (P^*^)

Correlation between the active and the controls (P^**^)

Correlation between the inactive and the controls (P^***^)

Data are expressed as mean ± SD

## Discussion

Although the pathogenesis of thrombosis in BD is not completely clear, it is generally accepted as the endothelial dysfunction caused by vasculitis as evidenced by increased serum thrombomodulin levels.[13] Hyperhomocysteinemia is a well-known risk for the development of thrombosis. Its role as a risk factor for thrombosis in BD has not fully investigated. In this study, we excluded other diseases and factors that cause hyperhomocysteinemia such as vitamin B12/folate deficiency, hyperlipidemia, diabetes mellitus, psoriasis, chronic hepatitis, chronic alcoholism, renal failure, and pregnancy. We studied serum Hcy concentration in BD with and without thrombosis and compared it with the concentration in controls. It was found that consistent with others’ findings.[13–16], the mean serum Hcy concentration was significantly higher in patients than in the controls of our study. It has been found in our study as well as a study by Aksu *et al*.[13] that the mean serum Hcy concentration was significantly higher in thrombotic than in nonthrombotic BD patients. The exact mechanism by which hyperhomocysteinemia causes vascular disease and/or thrombosis is not known. However, there are deleterious effects of hyperhomocysteinemia on endothelial cells, causing endothelial cell damage and increased oxidation stress.[17] On the other hand, Hcy-mediated vascular disease/thrombosis occurs via interference with coagulation mechanisms. Hcy inhibits the expression and activation of thrombomodulin which is a cofactor for protein C activation. Hcy also suppresses the anticoagulant action of antithrombin III.[18] On the other hand, other studies reported that hyperhomocysteinemia is not related to any thromboembolic evidence.[19,20]

It must be kept in mind that hyperhomocysteinemia may be also caused by genetic defects and/or by a deficiency of the vitamins (B12, B6, and folic acid) involved in homocysteine metabolism. It is evidenced by the study of Eichinger[21] who reported low vitamin B6 levels in association with the increased risk of a first venous thrombosis. As hyperhomocysteinemia is an independent risk factor and because folic acid and vitamin B12 supplement can lower the risk and incidence of hyperhomocysteinemia, hyperhomocysteinemia is a reversible risk factor, and thrombosis in BD can be preventable.[13]

BD is characterized by endothelial dysfunction, neutrophil hyperfunction, and the overproduction of reactive oxygen species (ROS).[8] Endothelin-1 (ET-1) is a vasoconstrictor peptide that is released from vascular endothelial and synovial cells.[22] In the present study, there were significant increases in plasma ET-1 concentrations in active BD patients compared to those in the controls, whereas there were no significant differences between ET-1 concentrations in the inactive BD patients and the controls. These observations are supported by other studies as well.[23,22,14,9] It is not known whether elevated ET-1 levels are a direct result of its increased synthesis from injured vascular endothelial cells or whether its high concentrations may be responsible, along with other factors, for both vascular and articular complications in patients with BD.[22] One study attributed the increase of serum ET-1 levels in BD to increased secretion or leakage of this mitogen from injured vascular endothelial cells.[23] It also stated that ET-1 may play an important pathogenetic role in the development or progression of vasculitis that is common in BD.[23] Another study reported an overproduction of ET-1 by the respiratory tract and in the serum of patients with pulmonary involvement of BD that may be contributed to the functional and morphological abnormalities of the vasculature that are associated with the disease.[24]

Nitric oxide (NO) is a reactive nitrogen intermediate that plays a key role in the pathogenesis of many inflammatory and autoimmune skin diseases. NO is one of the free radicals that is released in oxidative stress. Excess NO causes lipid peroxidation, cellular dysfunction, and death.[25] It is an important molecule for the vascular system. The proinflammatory cytokines are potent inducers of leptin. NO from endothelial cells is an important mediator of immunity and inflammation that induces endothelial vasorelaxation and the inhibition of platelet adhesion. It is enzymatically synthesized from L-arginine, a reaction that is catalyzed by nitric oxide synthase (NOS).[14]

The pathogenesis of BD may be related to the excessive production of ROS, activated neutrophils, and T lymphocytes.[9] It was reported that neutrophil activation may play an important role in the pathogenesis of BD as the main source of oxidative stress through protein oxidation, and that chlorinated oxidants of neutrophil origin may lead to oxidative stress, notably protein oxidation. Neutrophils are implicated in the pathogenesis of BD; the presence of *in vivo* preactivated neutrophils in BD has been reported.[26]

The present study showed a significant increase of serum nitrite levels in BD compared to the controls and also showed its correlation with disease activity; these results have been supported by other studies.[27,1,28] Duygulu *et al*. reported an increase of synovial NO levels in active patients that probably reflected a nonspecific inflammatory process of the synovium and, therefore, a resultant arthralgia and arthritis as a common finding of BD.[1]

In contrast, Aydin *et al*. reported that serum NO levels are not significantly changed in patients with BD and its subtypes,[29] whereas others reported decreased NO levels in the serum of BD patients.[30,16] Orem *et al*.[30] reported decreased production of nitrite and nitrate as indicators for NO in patients with BD and its critical biological activities that may be relevant to pathological events in the active period of the disease. Ozkan ***et al***[16] reported a high prevalence of hyperhomocysteinemia and its deleterious effects on the pathology of BD by decreasing NO levels and interfering with the immune system.

The results of routine investigations reported in literature are often within the normal range in BD. During an obvious flare of the disease, levels of acute phase reactants such as CRP and ESR may be moderately elevated.[31] All of these were confirmed by the findings of the present study.

## Conclusion

As hyperhomocysteinemia is a reversible risk factor since the thrombosis in BD can be preventable, another further study is recommended to evaluate the possibility to reduce the risk of idiopathic venous thrombosis in BD.
